# Electrothermally Driven Hydrogel-on-Flex-Circuit Actuator for Smart Steerable Catheters

**DOI:** 10.3390/mi11010068

**Published:** 2020-01-08

**Authors:** Madeshwaran Selvaraj, Kenichi Takahata

**Affiliations:** Department of Electrical and Computer Engineering, University of British Columbia, Vancouver, BC V6T1Z4, Canada; madeshwaran.selvaraj@alumni.ubc.ca

**Keywords:** medical catheters, steerable tip, electrothermal actuator, thermoresponsive hydrogel, flex-circuit microfabrication

## Abstract

This paper reports an active catheter-tip device functionalized by integrating a temperature-responsive smart polymer onto a microfabricated flexible heater strip, targeting at enabling the controlled steering of catheters through complex vascular networks. A bimorph-like strip structure is enabled by photo-polymerizing a layer of poly(*N*-isopropylacrylamide) hydrogel (PNIPAM), on top of a 20 × 3.5 mm^2^ flexible polyimide film that embeds a micropatterned heater fabricated using a low-cost flex-circuit manufacturing process. The heater activation stimulates the PNIPAM layer to shrink and bend the tip structure. The bending angle is shown to be adjustable with the amount of power fed to the device, proving the device’s feasibility to provide the integrated catheter with a controlled steering ability for a wide range of navigation angles. The powered device exhibits uniform heat distribution across the entire PNIPAM layer, with a temperature variation of <2 °C. The operation of fabricated prototypes assembled on commercial catheter tubes demonstrates their bending angles of up to 200°, significantly larger than those reported with other smart-material-based steerable catheters. The temporal responses and bending forces of their actuations are also characterized to reveal consistent and reproducible behaviors. This proof-of-concept study verifies the promising features of the prototyped approach to the targeted application area.

## 1. Introduction

Medical catheters are flexible tubes with different tip shapes used by surgeons to access a target location inside the human body [1]. They play a crucial role in the treatment of vascular and cardiovascular diseases, as well as many other surgical procedures [2]. With the help of X-ray fluoroscopy and magnetic resonance imaging, the distal tips of the catheters are controlled by moving their proximal ends outside the body [3,4]. These devices consist of a working channel typically used for administering fluids, inserting various micro tools, and/or controlling a guide wire that offers extra steering support, device tracking, and aids in catheter replacements [5]. The current catheters available on the market have fixed tips, which fact poses a variety of practical challenges. Surgeons are required to be highly skilled in guiding the catheters manually, and they need to use different catheters with different tip shapes to navigate through complex branch bifurcations to reach a target site [6,7,8,9]. For example, branch angles of the human coronary arteries can vary from 32° to 124° [10,11], making catheter navigation challenging. As such, the use of these devices can injure the inner walls of the vessel because of the friction produced by forcing a fixed tip to bend at a branch bifurcation and steer it in an intended direction [4,11]. Accordingly, there have been many efforts in realizing active “smart” catheters equipped with miniaturized actuators to help surgeons perform controllable and guided catheterization procedures [12]. 

Different approaches to this goal have been reported [13,14,15,16]. One promising approach that has been widely investigated is to use responsive “smart” materials as the actuator element aiming to achieve greater bending controllability, simpler system configurations, and miniaturization of the active tip [17]. The responsive materials that have been used in this area include shape-memory alloys [16,18,19,20], shape-memory polymers [21,22,23], conjugated polymers [24,25] and ionic polymer–metal composites [26,27]. Although these materials provide potential options for controlling the catheter tips, various practical problems are yet to be addressed, such as limited bending angles, response speeds, and complicated fabrication methods often caused by the material’s incompatibility with planar fabrication processes.

Hydrogels have been used in diverse biomedical applications [28,29,30]. Some of them are known as smart materials to swell and deswell in response to changes in specific environmental parameters, including temperature, pH, chemical, light and electric field [31,32,33]. Thermoresponsive hydrogels such as poly(*N*-isopropylacrylamide) (PNIPAM) [34] show a reversible phase transition at a lower critical solution temperature (LCST) above which they shrink and deswell the fluid. During the LCST phase transition, these hydrogels exhibit a shape memory effect to deform from a programmed-temporary shape to an original-permanent shape in response to temperature changes [35,36,37,38]. The molecular level transition mechanism of PNIPAM is well studied and documented [39,40,41]. One advantageous feature available with these hydrogels, including PNIPAM, is that they can be photo-patterned [42] to shape their structures in a manner compatible with planar lithographic processing [43,44,45]. Due to this feature, they have been actively studied for micro-electro-mechanical systems (MEMS) applications [46,47,48,49,50,51,52,53]. Moreover, PNIPAM hydrogel surfaces show good biocompatibility and are used extensively in tissue engineering research [54]. All of these characteristics have promoted the material’s applications in biomedical microdevice areas [55,56,57]. For example, hydrogel microstructures were used as miniaturized actuators stimulated by resistive [42,58,59] and magnetic [60] heating for drug delivery applications. The material compositions of PNIPAM can be modified to adjust the level of LCST [34,61], and tailor it for specific applications. For example, the LCST of PNIPAM was reported to shift to 37 °C and 45 °C by mixing 1 mol % and 2 mol % of 3-trimethylammoniumpropyl methacrylamide [62]. Similar shifting was also approached through copolymerization with the *N*,*N*′-diethylacrylamide monomer [61].

**Figure 1:**
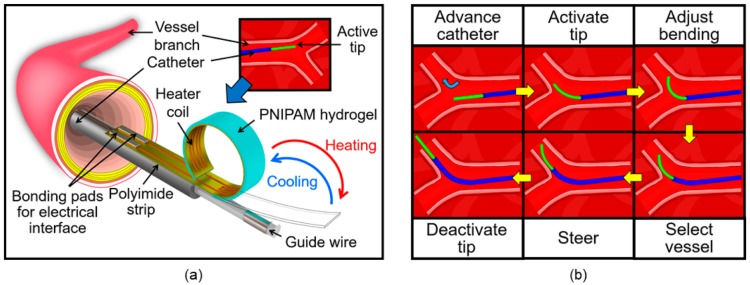
Conceptual diagrams of the steerable smart catheter tip: (**a**) Device structure and operation (illustrated for an example in blood vessel application); (**b**) an illustration on how the smart tip aids in maneuvering to an intended vessel branch while navigating through the human body.

This study explores an alternative path to develop a smart catheter tip using PNIPAM hydrogel as the active material. Figure 1a illustrates the device structure, in which the PNIPAM layer is integrated with a flexible strip of microfabricated heater that is fixed at the tip of a conventional catheter. The active-tip device provides the catheter with a steering ability for a wide range of bending angles, enabling the controlled maneuvering of the catheter to a target vessel branch (Figure 1b). This active functionality is aimed to mitigate or eliminate the need for multiple fixed-tip catheters during a catheterization procedure. This in turn eases the procedure, while substantially shortening the surgical time, minimizing the navigation skills required by surgeons, and reducing the risk of damaging vessel tissues during the intervention. The present work focuses on the experimental characterization and demonstration of microfabricated proof-of-concept prototypes to verify the effectiveness of the proposed device approach following our preliminary study [63]. The device configuration, operational principle, fabrication process and characterization results are discussed in the following sections.

## 2. Principle and Design

**Figure 2:**
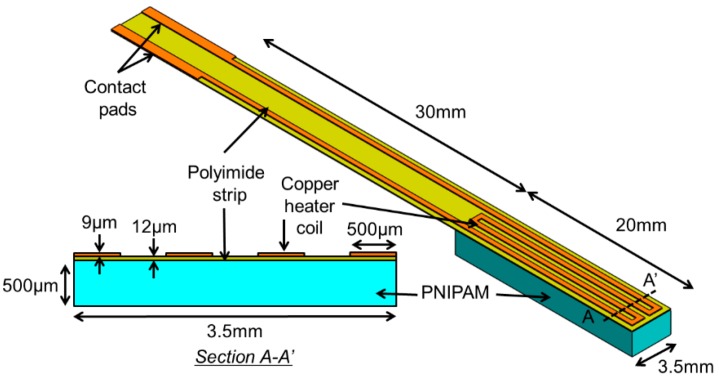
Device design showing the flexible active strip integrated with a poly(*N*-isopropylacrylamide) (PNIPAM) hydrogel layer and a micropatterned planar heater at the free end of the strip.

At temperatures lower than LCST, the polymer chain structures of PNIPAM hydrogel forms hydrogen bonds with water molecules and becomes swollen by absorbing water. Above the threshold temperature, the hydrogen bonds in the polymer break and shrink the material by releasing water. This phase transition can be electrically controlled by coupling the material with a heater element, in which the electrical energy applied to the heater is converted into thermal energy that triggers the polymer to produce mechanical displacement and force. Exploiting this mechanism, the active-tip device is designed to have a bimorph-like structure comprised of a flexible polymer strip embedded with a micropatterned, planar-coil heater made of copper and a layer of PNIPAM hydrogel with an approximate thickness of 500 μm upon swelling (Figure 2). The active strip is shaped to have a total length of 50 mm with a 20 × 3.5 mm^2^-sized heater on one side of the strip’s free end. A flat PNIPAM layer is formed on the heater region of the other side of the strip through a photo-polymerization process. When placed in water, the PNIPAM layer on the strip swells, and then fully curls in its base/cold state due to the bimorph effect. Activating the heater, the temperature of the hydrogel is raised to exceed its LCST, causing the polymer to shrink, which then induces a tensile stress to force the strip to straighten (Figure 1a). The bending angle of the active tip is controlled by varying the amount of electric power applied to the heater, and thus its temperature. This feature allows a surgeon to freely select a suitable branch and control the catheter to a target location by steering through the vascular system containing branches with many different bifurcation angles.

## 3. Device Fabrication

**Figure 3:**
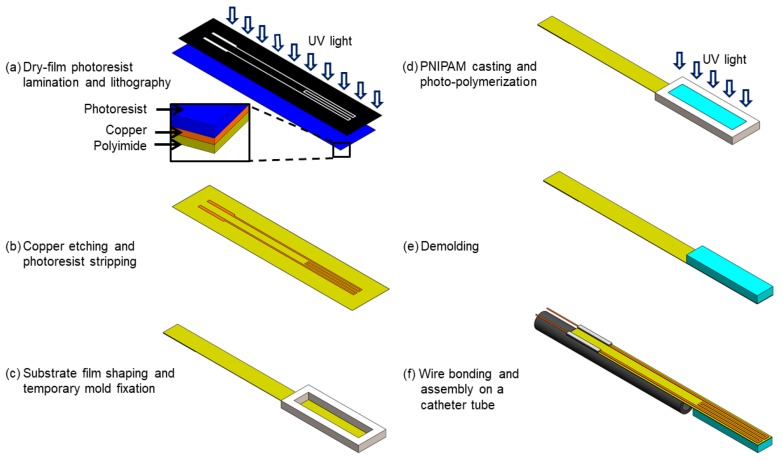
Process steps developed for the fabrication of the active catheter tip device.

The flexible heater strip is microfabricated through a flex-circuit process [46]. A single-sided, copper-clad polyimide film (Pyralux AC 091200EV, Dupont., Wilmington, DE, USA) with a thickness of 12 μm is used to create the heater element using its 9-μm-thick clad layer on the polyimide film that is shaped to be a strip. First, a 17-μm-thick dry-film negative photoresist (LF106, MacDermid Inc., Waterbury, CT, USA) is laminated on the clad side of the film using a hot-roll laminator (XRL-120, Western Magnum Co., El Segundo, CA, USA) maintained at 100 °C, and then patterned using a mask aligner (PLA-501F, Canon, Tokyo, Japan) to form the etch mask for the heater coil pattering after development (Figure 3a). The copper layer is then wet etched to form the built-in heater with its contact pads, followed by stripping the photoresist (Figure 3b). The strip is shaped out of the base polyimide film by laser cutting or other methods (Figure 3c). A precursor solution of photosensitive PNIPAM containing 2-mol % crosslinker [62] is synthesized by mixing *N*-isopropylacrylamide (monomer), *N*,*N*′-methylene bisacrylamide (crosslinker) and 2,2′-dimethylsulphoxide (photoinitiator) in dimethylsulphoxide (solvent), with a composition of 5.72 g, 157.6 mg, 377.2 mg and 10 mL, respectively. The metal mold with a cavity size of 20 × 3.7 mm^2^, an area close to that of the heater, is secured on the heater location by pressurizing the mold onto the polyimide side of the strip to cast the hydrogel solution. The mold is created out of an 850-μm-thick aluminum sheet using precision water-jet cutting and precisely aligned with the heater pattern under a microscope before fixing. Casting is carefully processed to inject 40 μL of the precursor solution into the mold using a precision micro syringe.

**Figure 4:**
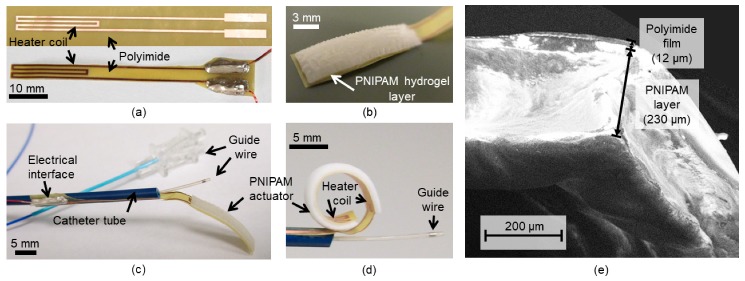
Optical images of fabrication results: (**a**) (Upper) flexible strip of the tip device showing the patterned heater element, and (Bottom) the shaped tip device with electrical interface (copper wires bonded to the contact pads); (**b**) PNIPAM layer integrated on the other side of the heater; (**c**) active tip device assembled on a commercial catheter tube (7.8-french Pebax^®^ 7233, Zeus Inc., Orangeburg, SA, USA) with a guide wire (RCF-7.0-35 check Flo Introducer, Cook Medical, Bloomington, IN, USA) inside its channel; (**d**) final curled shape of the device tip after swelling; (**e**) Scanning electron microscope (SEM) image of the device edge (captured using Sigma 500, Zeiss Sigma, Carl Zeiss NTS Gmbh, Oberkochen, Germany).

Next, the cast solution in the mold is flood exposed to ultraviolet light (395-nm wavelength) to trigger cross-linking of the PNIPAM layer (Figure 3d), and then the mold is removed (Figure 3e). The particular polyimide–hydrogel interface is observed to exhibit an adhesion high enough to prevent delamination upon demolding and subsequent full bending of the strip post swelling. The fabricated active tip device remains nearly flat after this photo-polymerization step (as shown in Figure 4b). The electrical interface to the device is established by bonding copper wires to the contact pads using a conductive adhesive. The inactive part of the device is then wrapped and bonded around a medical catheter tube using a medical-grade epoxy (Cyberlite U303, Cyberbond, Frankfort, IL, USA) (Figure 3f). Finally, the device is immersed in de-ionized (DI) water for 30 min to allow the hydrogel to fully swell, expand and deform the flat structure into a curled shape. Figure 4 shows different states of the fabricated prototype. Scanning electron microscope (SEM) imaging of the PNIPAM layer on the polyimide strip indicates that the dried layer thickness was ~230 μm and relatively uniform (Figure 4e). After being fully swelled with water, the layer thickness was measured (using a stylus profilometer; DektakXT, Bruker Co., Billerica, MA, USA) to reach ~500 μm showing ~2.2× volume expansion. This high uptake of water accounts for the large deformation (curling) of the device tip as seen in Figure 4d.

## 4. Results and Discussion

### 4.1. Characterization of PNIPAM Synthesized on the Flexible Strip

**Figure 5:**
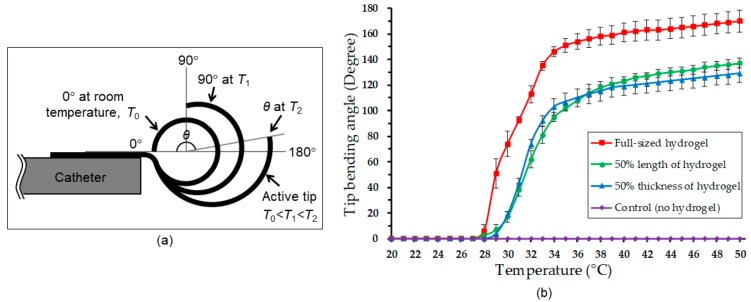
Thermomechanical characterization: (**a**) Illustration of bending angle measurement based on optical imaging showing three different states of the device tip at different ambient temperatures; (**b**) the bending angles of the fabricated prototypes with different hydrogel dimensions (20 mm or 10 mm length, 0.5 mm or 0.25 mm thickness) measured in a deionized (DI) water bath while varying water temperature up to 50 °C.

As a preliminary test of the fabricated prototypes, their thermomechanical responses were characterized in a bath of DI water by varying its temperature. In this experiment, the device prototype was horizontally secured on top of a protractor in the water bath. The original, fully curled device at room temperature was carefully aligned so that its tip was positioned at 0° of the protractor as illustrated in Figure 5a. The bath was heated from room temperature (22 °C) to 50 °C using a hot plate during which the immersed device was imaged at every degree Celsius change in water temperature. 

The captured images were analyzed to track angular changes made in the device tip. The bending angle was calculated by measuring the difference in angle between the initial location (0°) and a newly deformed position of the tip (Figure 5a).

To observe the PNIPAM’s structural dependence of the response, the following four different samples were tested: The device with the full length (20 mm) and the full thickness (0.5 mm under swelling) of the hydrogel, the device with half the length (10 mm) and the full thickness of the hydrogel, the device with the full length and half the thickness (0.25 mm under swelling) of the hydrogel, and a control device with no hydrogel. The reduced length of the hydrogel layer was defined using a different cast mold with the corresponding length, and the thickness of the layer was halved by adjusting the casting volume of the hydrogel solution.

The control device tip remained flat with no deformation observed throughout the test, while the hydrogel-integrated prototypes showed varying tip deformations and angles depending on the water temperature and the device types (Figure 5b). These results confirmed that the deformation of the device tip only originated in the active material. All the hydrogel-integrated prototypes exhibited phase transition temperatures (approximately 28–29 °C) close to the expected LCST of the particular PNIPAM used in the device [62]. As the water temperature rose, the device with the full hydrogel dimensions gradually uncurled to straighten the tip, reaching a bending angle of 170° when temperature was raised to 50 °C. This bending amount is found to be the largest among other reported smart-material-based catheters (13–155°) [13,14,15,16]. As also can be seen, the devices with the halved length or thickness of the hydrogel layer showed actuations with reduced bending angles as expected. It is noticeable that the bending responses of these two devices were similar. This might be a reasonable outcome, as the volume of the hydrogels used in the two are approximately identical. From these results, the device with the defined design was verified to provide the largest bending, and was thus used for the subsequent tests.

### 4.2. Electrothermal Response

**Figure 6:**
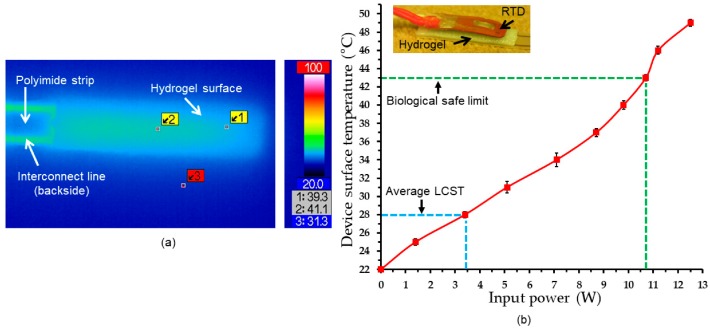
Electrothermal characterization of the active tip: (**a**) An infrared (IR) image showing surface temperatures of the swelled PNIPAM hydrogel in air; (**b**) device surface temperatures vs. input power to the built-in heater measured in water (insert image showing the device attached with the resistance temperature detector (RTD) used for the measurement).

Prior to the actuation test, electrothermal behaviors of the built-in heater and its heat transfer to the integrated hydrogel layer were evaluated. For this purpose, the heater coil with the swelled PNIPAM on the device was activated, first in air, under an infrared (IR) camera (VarioCAM HiRes Research 1.2 Mega, Jenoptik, Germany). Figure 6a shows an IR image of the PNIPAM side of the activated device (with an input power of 0.57 W). The image verified a relatively uniform heat distribution, with a maximum temperature variation of ~2 °C, over the entire region of the active hydrogel layer. 

Next, electrothermal responses of the device in water medium were evaluated by probing and tracking their surface temperatures. This under-water temperature measurement used an insulated resistance temperature detector (RTD; S667PDZ40AC, Minco Products, Inc., Minneapolis, MN, USA). The RTD was directly fixed on the hydrogel surface of the device (as in the insert image of Figure 6b) and placed in a DI water bath at room temperature. The built-in heater was powered with varying levels each for 18 s after which the RTD reading was recorded to evaluate the steady-state temperature of the device. Figure 6b shows the measured temperatures as a function of input power, showing almost linear increases in temperature overall (e.g., + 9 °C and + 18 °C with powers of 5.1 W and 10 W, respectively). A power range of 3.5–4 W was required to reach 28–29 °C in temperature, the measured LCST range of the device. As the power fed to reach the LCST level theoretically does not contribute to the actuation, a possible approach to reduce power consumption in the device would be to adjust the LCST level closer to (but slightly above) the ambient temperature, i.e., the human body temperature for real application setting of the device. This experiment also revealed the maximum power that maintained the device temperature within a safe level that does not cause thermal damage to tissue (~43 °C or less) [64] was 10.7 W (as indicated in Figure 6b).

**Figure 7:**
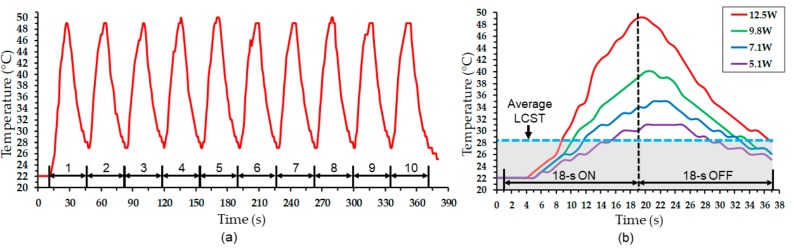
Temporal electrothermal response of the device evaluated by iterative cyclic powering, showing (**a**) the result of ten powering cycles with a fixed power and (**b**) a close-up of single cycle measured with varying input powers.

Cyclic powering of the heater (18 s on and 18 s off, with 12.5 W power) showed a consistent and reproducible response of the device in both heating and cooling cycles, reaching a peak temperature of ~49 °C upon heating and cooled down to a base temperature of ~28 °C after turning off the heater (Figure 7a). The PNIPAM of the device under test was visually observed to repeatedly deswell and swell during each cycle. Figure 7b shows detailed responses of the device in one powering cycle with four different power levels ranging from 5.1 W to 12.5 W. The graph also indicates the measured level of LCST above which mechanical deformation of the device could occur (but was constrained with the RTD element fixed to the device in the current test). It can be seen that higher input powers made the hydrogel stay at temperatures above the LCST level, or the deswelled/straightened state, for a longer time before the temperature reduced back to the threshold level. The plots also indicate that the temporal response was somewhat increased by raising the power level. For all the cases, the hydrogel was observed to hold certain amounts of heat at the end of cooling cycle, suggesting that heat dissipation from the device took more time than the set cooling time. It is worth noting however that cooling is expected to be faster when present in a flow of ambient medium (e.g., blood flow), compared to the case with no effect of forced convective heat transfer like the current test.

### 4.3. Actuation Test Results 

**Figure 8:**
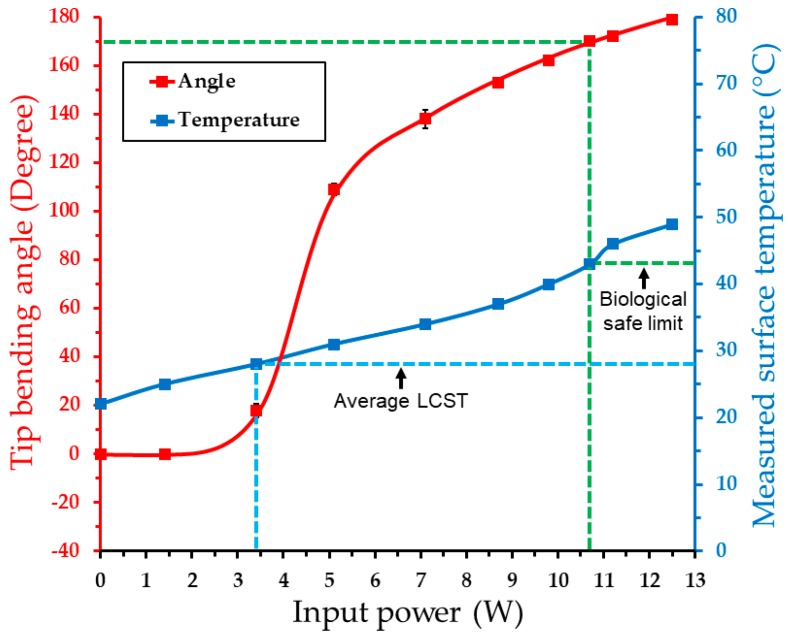
Measured angular displacement of the activated device as a function of the applied power to the embedded heater (measured device surface temperature from Figure 6b included for comparison).

To assess the device’s angular displacements induced by activation of the embedded heater, the prototypes were placed and actuated on top of a protractor in a DI water bath. For this, the operated device was video/image recorded to quantify the transient and steady-state tip bending angles as functions of time and input power for post-measurement processing. The steady-state displacement of the device was quantified by recording its tip bending angle at the end of 18 s activation duration. Figure 8 plots the device’s bending response measured with varying input powers, together with the measured electrothermal response (as shown in Figure 6b) for comparison. The results clearly indicate the LCST phase transition of the actuator via electrical activation, which triggered a significant bending at a power of 3.4 W. From the previous test, this power level was characterized to set the integrated PNIPAM temperature at ~28 °C, which matches well with the measured LCST level (Figure 5). After the initial large displacement, the device showed nearly linear increases of bending angle at a reduced rate with input powers up to 12.5 W that led to the maximum angle of 179°, as shown. The bending angle within the biologically safe temperature was observed to be 170°. 

It should be noted that this amount of available bending is expected to decrease when the LCST level is shifted to be above the body temperature. As seen in the graph, the major displacement occurs right above the LCST. If a hypothetical setting that the LCST is adjusted around 38–39 °C is considered, the temperature window that the device could be safely operated is approximately 4–5 °C. This, in combination with the results in Figure 8, suggests that the device could still provide ~120° of bending angle. This bending amount is still substantially greater than those reported with most of other smart-material active catheter devices (representing their inherent capabilities, with unknown consideration in biocompatible operation). In addition, these temperature and actuation ranges could be made larger if certain packaging (for thermal insulation) is implemented in the device. Nevertheless, this test result demonstrated that the fabricated device provided an expected controllability of tip bending angle via electrical powering, with bending amounts potentially suited for use in human coronary arteries covering a wide range of their branch angles of up to 124° [10,11].

**Figure 9:**
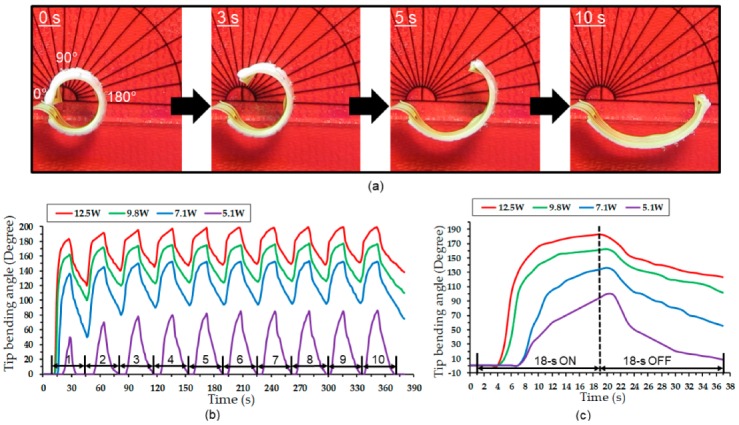
Temporal bending response of the device: (**a**) Optical images of transitional shapes of the activated device (placed over a protractor) showing the maximum bending angle of >170° at 10 s of powering (at 12.5 W); iterative cyclic powering (18 s on and 18 s off) showing (**b**) the results of ten powering cycles with varying input powers and (**c**) a close-up of single cycle.

Figure 9a shows a series of transient images of the device activated with the maximum power used. It can be seen that the tip resulted in a bending angle of 172° after 10 s, in which >80% of the displacement was completed after half (5 s) of the duration. The temporal bending behavior of the device was further evaluated through cyclic testing using four different input powers (identical to those used in the electrothermal tests discussed earlier). Figure 9b plots the measured bending angles for ten cycles of activation and deactivation, each lasting 18 s. As displayed in the results, the device exhibited stable and reproducible temporal responses after the first or second cycle. The maximum bending amounts observed with different input powers approximately matched the steady-state results from Figure 8, except for the case with the lowest power (of 5.15 W; ~110° steady-state angle vs. ~85° cyclic angle). This exception is presumably related to the used heating time, which was likely not sufficient for the active tip to produce the maximum possible temperature/bending due to the time lag to reach the LCST level. Detailed responses of the device in one powering cycle are shown in Figure 9c. For activation with 12.5 W power, the tip was observed to attain most of its maximum bending amount relatively quickly, in ~8 s of activation, after which the displacement gradually stabilized to reach the maximum angle of ~200°. To the best of the authors’ knowledge, this recorded bending angle is among the largest in the results of reported steerable catheters using various actuation mechanisms [13] or comparable to 220° reported for a large-scale tendon catheter system [65]. The case with 9.8 W power followed a similar trend (with a slightly lower maximum angle). With the lower powers (7.1 W and 5.1 W), the time lags to reach the LTSC were apparent (and for the 5.1 W case, this led to the lower maximum bending angle compared with the result in Figure 8, as noted above). As also apparent from Figure 9c, returning/curing motions during the cooling cycles were slower than the straightening motions during the heating cycles due to relatively slow heat dissipation from the hydrogel/heater, as also noted earlier.

**Figure 10:**
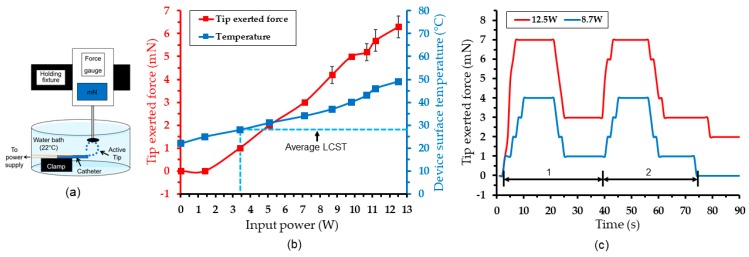
Actuation force characterization: (**a**) Measurement set-up used; (**b**) measured forces as a function of input power (measured device surface temperature from Figure 6b included for comparison); (**c**) temporal force response measured with two different input powers.

The actuation force exerted by the prototype was characterized using the experimental set-up illustrated in Figure 10a. The device was again placed in a DI water bath maintained at room temperature while the tip of the device (under its cooled/curled state) was placed against the probe tip of a digital force gauge (DS2-1, Imada Inc., Northbrook, IL, USA; 1-mN resolution). The activation of the device straightened the tip to exert a force onto the gauge, which was recorded as a function of input power at the end of 18 s of the powering cycle. Figure 10b plots the measured average forces together with the corresponding steady-state device temperatures (from Figure 6b) for comparison. 

The force response shows almost a linear trend with the powers up to 12.5 W, at which the recorded maximum force was 7 mN. A detectable force was seen at a 3.4 W power. This power level corresponds to 28 °C of the device temperature, which matches very well with the measured LCST of the used PNIPMAM. These results suggest that the force generated by tip can be well controlled by the amount of power applied to the device. Figure 10c shows sample temporal responses of the tip force measured by activating the device with the previously used cycle times (18 s on and 18 s off) at two different input powers. It can be seen that the device tip exerted a maximum force (of 7 mN at 12.5 W and 4 mN at 8.7 W) within 8 s of activation, after which it stabilized for the rest of the heating cycle. The tip was recorded to maintain small residual forces (3 mN at 12.5 W and 1 mN at 8.7 W) at the end of the off-cycle time. This is consistent with the observed actuation behavior that exhibited slower motions during the off cycle than the on cycle, as previously discussed. 

## 5. Conclusions

A smart catheter-tip device based on a bimorph-like structure has been designed, microfabricated, and demonstrated towards achieving more efficient and safer catheterization procedures. The device was enabled by integrating a thermoresponsive hydrogel on top of a heater-embedded flexible polyimide strip, showing a controlled bending of the tip structure with the amount of input power fed to the heater. The prototypes were developed through the flex-circuit manufacturing technology in combination with a PNIPAM photo-polymerization process to offer a potential path to a low-cost production of the device with its simple design. The active tip was observed to achieve uniform heat transfer to the hydrogel layer, which exhibited a thermal phase transition within a threshold temperature level that matched well with the expected LCST of the PNIPAM used. Electrothermally stimulating the active tip, the microfabricated prototypes were demonstrated to produce large angular displacements, representing a remarkable improvement from those reported with similar devices based on smart materials, while showing stable cyclic operations in an experimental setting. These results encourage the further development and refinement of the device design for quick and safe steering through complex branch bifurcations. Future work will encompass the optimization of the hydrogel’s thermal response with an adjusted LCST level (with respect to human body temperature), as well as device design, including further miniaturization and biocompatible packaging of the device towards testing in flow-loop and in vivo models.
